# MiR-124-3p inhibits tumor progression in prostate cancer by targeting EZH2

**DOI:** 10.1007/s10142-023-00991-8

**Published:** 2023-03-08

**Authors:** Bao-feng Song, Li-zhe Xu, Kun Jiang, Fan Cheng

**Affiliations:** Department of Urology, Renmin Hospital of Wuhan University, Wuhan, 430060 Hubei Province People’s Republic of China

**Keywords:** Proliferation, Invasion, Apoptosis, MiR-124-3p, EZH2, Prostate cancer

## Abstract

Prostate cancer (PCa) is widespread cancer with significant morbidity and mortality rates. MicroRNAs (miRNAs) have been identified as important post-transcriptional modulators in various malignancies. This study investigated the miR-124-3p effect on PCa cell proliferation, infiltration, and apoptosis. EZH2 and miR-124-3p expression levels were measured in PCa tissues. PCa cell lines DU145 and PC3 were transfected with miR-124-3p inhibitors or analogs. EZH2 and miR-124-3p linkage was validated by conducting the luciferase enzyme reporter test. The cell viability and apoptosis were assessed by flow cytometry and MTT test. Cell movement was noted during infiltration using transwell assays. EZH2, AKT, and mTOR contents were assessed using qRT-PCR and western blotting. In clinical PCa specimens, miR-124-3p and EZH2 contents were inversely correlated. Further research has demonstrated that EZH2 is the miR-124-3p direct target. Furthermore, miR-124-3p overexpression reduced EZH2 levels and lowered cell viability, infiltration, and promoted cell death, whereas miR-124-3p silencing had the opposite effect. Overexpression of miR-124-3p decreased the phosphorylation level of AKT and mTOR, whereas miR-124-3p downregulation produced the opposite result. Our findings depict that miR-124-3p prevents PCa proliferative and invasive processes while promoting apoptosis by targeting EZH2.

## Introduction

Prostate cancer (PCa) is highly prevalent and affects millions of men worldwide, especially in regions with a high human development index (Hoter et al., 2019; Xu et al., 2022). PCa prevalence has steadily increased in China in recent years. Its diagnoses have been simply based on prostate-specific antigen (PSA) usage; however, its lack of specificity typically contributes to diagnostic errors. This contributes to several unnecessary and repeated prostate biopsies, including complications and overtreatment of clinically insignificant tumors (Zhang et al., 2019; Lomas & Ahmed, 2020; Díaz-Fernández et al., 2021). Although recent advancements in prostate cancer treatments, such as chemotherapy, surgical prostatectomy, androgen deprivation therapy (ADT), immunotherapy, and radiation therapy, have improved patient survival, the prognosis for prostate cancer patients remains poor and serious side effects persist. Prostate cancer, in its advanced stages, remains incurable (Chistiakov et al., 2018; Sur et al., 2019). Therefore, to improve clinical skills in prostate cancer patients’ diagnosis, treatment, and prognosis, it is necessary to elucidate prostate cancer-specific molecular mechanisms for development and progression.

EZH2 is a Polycomb Repressive Complex 2 (PRC2) enzyme that stimulates histone H3 lysine 27 (H3K27me3) trimethylation, resulting in transcriptional silencing. It is a prominent histone methyltransferase (HMTase) (Simon & Kingston, 2009; Holoch & Margueron, 2017; Blackledge et al., 2015). EZH2 has been revealed as an oncogene in a multitude of tumors (Duan et al., 2020; Lue & Amengual, 2018), including ovarian, breast, stomach, endometrial, and colon cancers (Pan et al., 2016). A recent study explored inhibiting prostate cancer development by targeting EZH2 (Yuan et al., 2020). Together, these findings imply that EZH2 is critical for PCa evolution.

Gene expression is controlled by microRNAs (miRNAs), which degrade messenger RNAs (mRNAs), inhibit protein production, and interact with large non-coding RNAs. MicroRNAs are highly conserved across species (Xie et al., 2018; Chan & Tay, 2018) and are involved in cell proliferative, apoptotic, differentiative, and invasive processes, as well as carcinogenesis, according to an increasing body of evidence. They play pivotal roles in cancer development and progression (Piatopoulou et al., 2017; Cao et al., 2016; Markopoulos et al., 2017; Nicoloso et al., 2009; Liu et al., 2017). Research has demonstrated that miRNAs in prostate cancer can act as oncogenes or tumor suppressors (Fabris et al., 2016; Daniunaite et al., 2017). miR-215-5p, for example, inhibits prostate cancer metastasis by targeting PGK1, whereas miR-671-5p increases prostate cancer metastasis by targeting the NFIA/CRYAB axis. miRNA-204-5p induces apoptosis in prostate cancer cells by targeting BCL2, and miR-92a expression is low in PCa cells and inhibits PCa cell viability and metastasis by targeting SOX4 (Lin et al., 2017). Despite this, the miR-124-3p biological impacts through PCa progression and the mechanisms that underpin them have yet to be identified.

The miR-124-3p quantity in PCa tumor tissues was inversely linked to EZH2, demonstrating that EZH2 directly affects miR-124-3p. These results confirmed that miR-124-3p has a regulatory role in PCa cell proliferation, infiltration, and death.

## Materials and methods

### Dataset analyses

TCGA database was used to obtain RNA-seq gene expression data and clinical data. Data were downloaded in the format of level 3 HTSeq fragments per kilobase per million (FPKM). TCGA data were used to analyze EZH2 levels in 26 human cancer types, 499 PCa tissues with 52 normal prostate tissues, and 52 PCa tissues with their paired neighboring normal prostate tissues. Correlation analyses of EZH2 were conducted using “ggplot2” in R. Kaplan-Meier plot was established, and a log-rank test was conducted with “survival” in R. ROC curve of diagnosis was created with R using the pROC package. Putative regulatory miRNAs of EZH2 were extracted from the Pic Tar, TargetScan, DIANA, and ENCORI databases.

### Clinical tissues

Wuhan University Renmin Hospital, the Medical Study Ethics Committee, authorized this research and was conducted under ethical management requirements. From 2019 to 2020, the urology department at Wuhan University Renmin Hospital provided 24 PCa tissues and non-malignant specimens. Prostate cancer and corresponding noncancerous tissues were obtained from 24 PCa patients, including 19 patients with primary tumor and 5 patients with metastatic tumor. Written consent was obtained from all participants in this research. Tumor node metastases (TNM) and categorization systems of the World Health Organization were used to determine tumor stage and grade. In two groups, the tissues were randomly categorized; the first was fixed with 4% PFA, and the second was preserved at − 80 °C.

### Cell lines and culture

PC3, LNCaP, and DU145, along with the human non-malignant prostate epithelial cell line RWPE1, were used in this study. The American Type Culture Collection obtained cell lines seeded into RPMI-1640 medium with 10% FBS under 5% CO_2_ and 95% O_2_ conditions.

### Cell transfection

The cells were multiplied using Lipofectamine™ 2000 platform (Invitrogen, USA) with miR-124-3p repressors, miR-124-3p analogs, or miRnegative controls (NCs), according to the manufacturer’s guidelines. The small interfering si-RNA for EZH2 (si-EZH2), EZH2 overexpression plasmid vector with pcDNA 3.1 and negative controls were purchased from Ribo Co., Ltd (Wuhan, China). The cells were cultured for two days following transfection before further treatment.

### Plasmid creation and luciferase enzyme reporter assays

EZH2-MUT (mutant-type) and EZH2-WT (wild-type) reporter plasmids were created by synthesizing and propagating the anticipated and mutated miR-124-3p target-binding sequences in EZH2 into a luciferase reporter. PCR was used to amplify the wild-type human EZH2 3’-UTR segment from PC3 cells, which contained the anticipated miR-124-3p target locations. The mutant EZH2 3’-UTR sequence was generated by overlap-extension PCR. Next, wild-type and mutant 3’-UTRs were sub-replicated into the psiCHECK-2 luciferase vector. Into 24-well culture plates, PC3 cells were seeded and co-transfected with miR-124-3p or an NC repressor using the Lipofectamine® 2000 system in the luciferase enzyme reporter studies. We extracted the cell transfects after 2 days. The luciferase enzyme activity evaluation was conducted utilizing Dual-Luciferase enzyme reporter assay platform.

### Immunohistochemical staining

For this operation, the tissues were fixed with 4% PFA, dried, and paraffin-embedded, then segmented at 4 μm thickness, and then infected overnight at 4 °C with rabbit polyclonal anti-EZH2 antibodies. After three PBS washes, all segments were infected with goat anti-rabbit IgG for 30 min at room temperature. Staining was observed using DAB and an Olympus BX50 light microscope.

### Western blotting

To purify total protein, we used RIPA lysis buffer containing PMSF, and to quantify it, we used the BCA kit. Equal protein quantities were fractionated on 10% SDS–PAGE gels and then transferred to PVDF membranes. After blocking, they were infected with primary antibodies against EZH2 (Abcam, UK; ab186006), p-mTOR (Abcam, UK; ab109268), mTOR (Abcam, UK; ab134903), GAPDH, and AKT (Abcam, UK; ab8805), p-AKT (Abcam, UK; ab38449) overnight at 4 °C incubation. After that, ECL (enhanced chemiluminescence) system kit was utilized for 1-h secondary antibody inoculation.

### RNA isolation and RT-qPCR

TRIzol® reagent was used to isolate RNA from clinical samples and PCa cells (Invitrogen, USA). A Takara RNA PCR kit synthesized cDNA from RNA (Takara Biotechnology, Japan). qPCR was performed on an ABI 7900 Real-Time PCR system using an SYBR Green mix kit from Applied Biosystems. U6 and GAPDH for miRNAs and mRNAs, respectively, were used to normalize miR-124-3p or EZH2 mRNA expression and compute miRNA and mRNA relative quantities, the 2^−∆∆Ct^ method was utilized. The oligonucleotide sequences are listed in Table 1.

**Table 1:** RT-PCR primer sequences

Genes	Primer sequence (5′-3′)
EZH2	F: GCCAGACTGGGAAGAAATCTG
	R: TGTGTTGGAAAATCCAAGTCA
miRNA-124-3p	F: CTCAACTGGTGTCGTGGAGTCGGCAATTCAGTTGAGGGCATTCA
	R: ACACTCCAGCTGGGTAAGGCACGCGGTGAATGCC
U6	F: ATACAGAGAAAGTTAGCACGG
	R: GGAATGCTTCAAAGAGTTGTG
GAPDH	F: TCATTTCCTGGTATGACAACGA
	R: GTCTTACTCCTTGGAGGCC

### Cell proliferation assay

An MTT assay was utilized to determine the miR-124-3p effects on cell viability. In 96-well plates, 2 × 10^3^ PCa cells were inoculated for 1, 2, 3, or 4 full days. A μmL MTT (5 mg/mL) were added to each well and infected in the plate at 37 °C for 4 h following the treatment. Then, we dumped the growing media and dispersed the formazan crystals using 150 μL DMSO. Using an ELX 800 microplate reader, the absorbance was measured at 490 nm.

### Cell apoptosis assay

The apoptosis assays were conducted using a FITC annexin V apoptosis detection kit. In 6-well plates, we injected 10^5^ PC3 cells per mL or 10^5^ DU145 cells per mL. Following that, the cells were incubated for 15 min in darkness at RT with nnexin V-FITC and PI, 5 mL each, before performing flow cytometry analysis (BD LSRII).

### Cell infiltration assays

Transwell chambers were used to investigate the PCa cell invasion ability (Corning Life Sciences). Cells (1 × 10^5^ cells were injected into the upper Matrigel-coated chamber in serum-free medium, while the lower chamber received a medium supplemented with 10% FBS. The upper chamber membrane was wiped away at 37 °C followed for 24-h incubation. The lower chamber cells were fixed and stained with 0.1% crystal violet for 30 min before imaging.

### Statistical analysis

R version 3.6.3 was utilized for all bioinformatics analyses. The data are depicted as the mean ± SD. Statistical differences were determined using a two-tailed Student’s *t*-test or *χ*^2^ test. ROC curve analysis was performed with the area under the curve (AUC) as the diagnostic accuracy index. Kaplan-Meier analysis was used to draw the survival curve. Every experiment was performed at least three times. Statistical analyses were performed using SPSS v2.0. *P* < 0.05 was regarded as statistically significant.

## Results

### EZH2 expression content in PCa and normal tissues

**Fig. 1:**
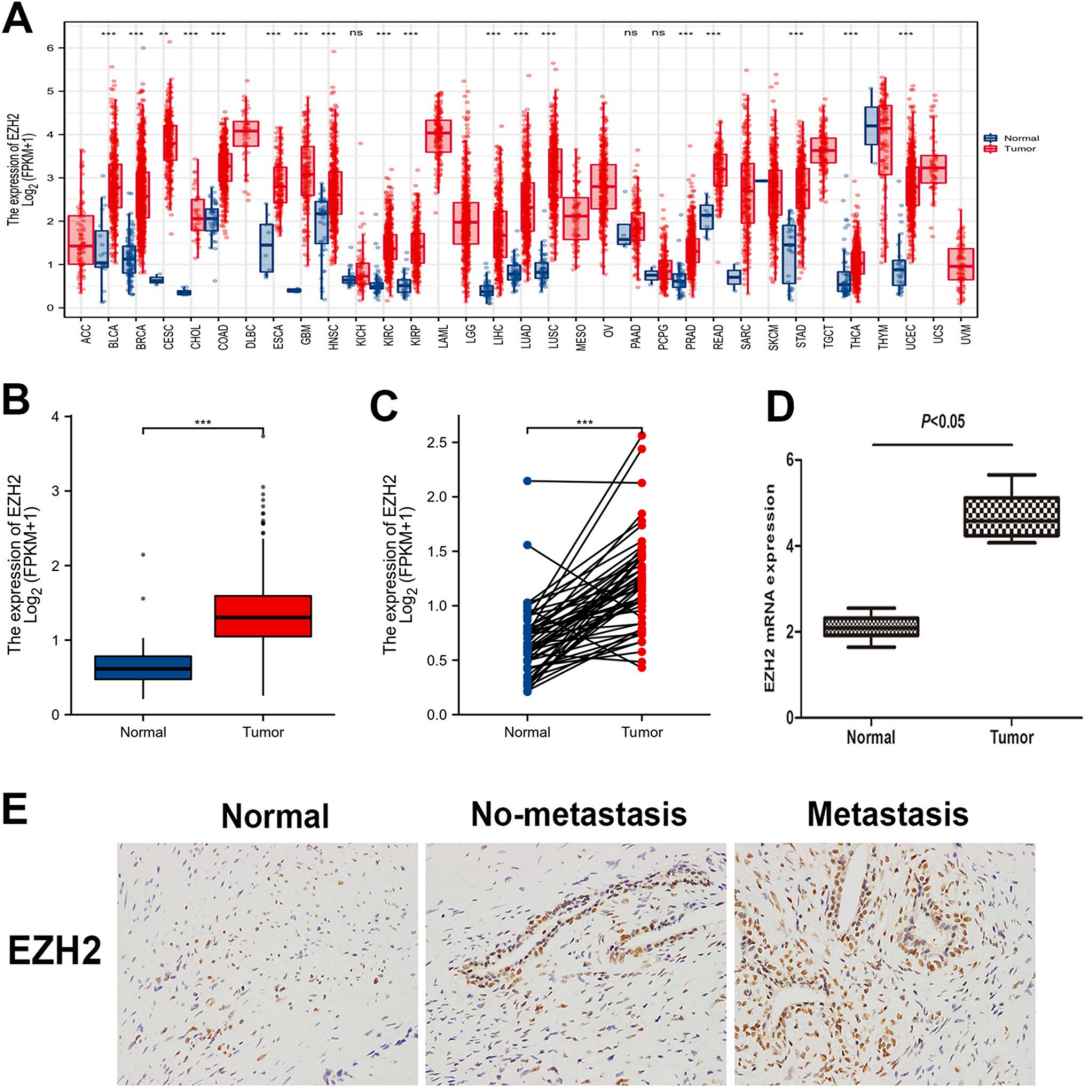
EZH2 expression content in PCa and normal tissues. **A** TCGA data illustrate that EZH2 is highly expressed in most cancer species. **B**, **C** Bioinformatics analysis of EZH2 expression levels in PCa (*n* = 499) and normal prostate tissues (*n* = 52) in TCGA database, the EZH2 levels were higher in PCa. **D** The qRT-PCR findings revealed that EZH2 was considerably more elevated in the PCa group than in the non-malignant group. **E** EZH2 immunohistochemistry in nonmetastatic PCa tissues (*n* = 5) and metastatic PCa tissues (*n* = 19) and benign prostate tissues (*n* = 24) (*p < 0.05; **p < 0.01; ***p < 0.001; ns, no significant difference)

EZH2 expression was analyzed using the Cancer Genome Atlas (TCGA) database. The TCGA data of PCa was downloaded from TCGA database which contained 499 tumor tissue samples and 52 normal prostate samples. The levels of EZH2 in most cancers were markedly upregulated relative to those in the control tissues (Fig. 1A). Moreover, EZH2 mRNA levels were considerably elevated in PCa tissues relative to adjacent normal tissues (Fig. 1 B and C). EZH2 expression was determined by immunohistochemical staining and qRT-PCR after analyzing 24 PCa tissue specimens paired with their neighboring non-malignant tissues (Fig. 1 D and E). Collectively, these data indicate that EZH2 expression was significantly increased in PCa tissues.

### Prognostic and diagnostic value of EZH2 in PCa patients in TCGA along with potential regulatory miRNAs of EZH2

**Fig. 2:**
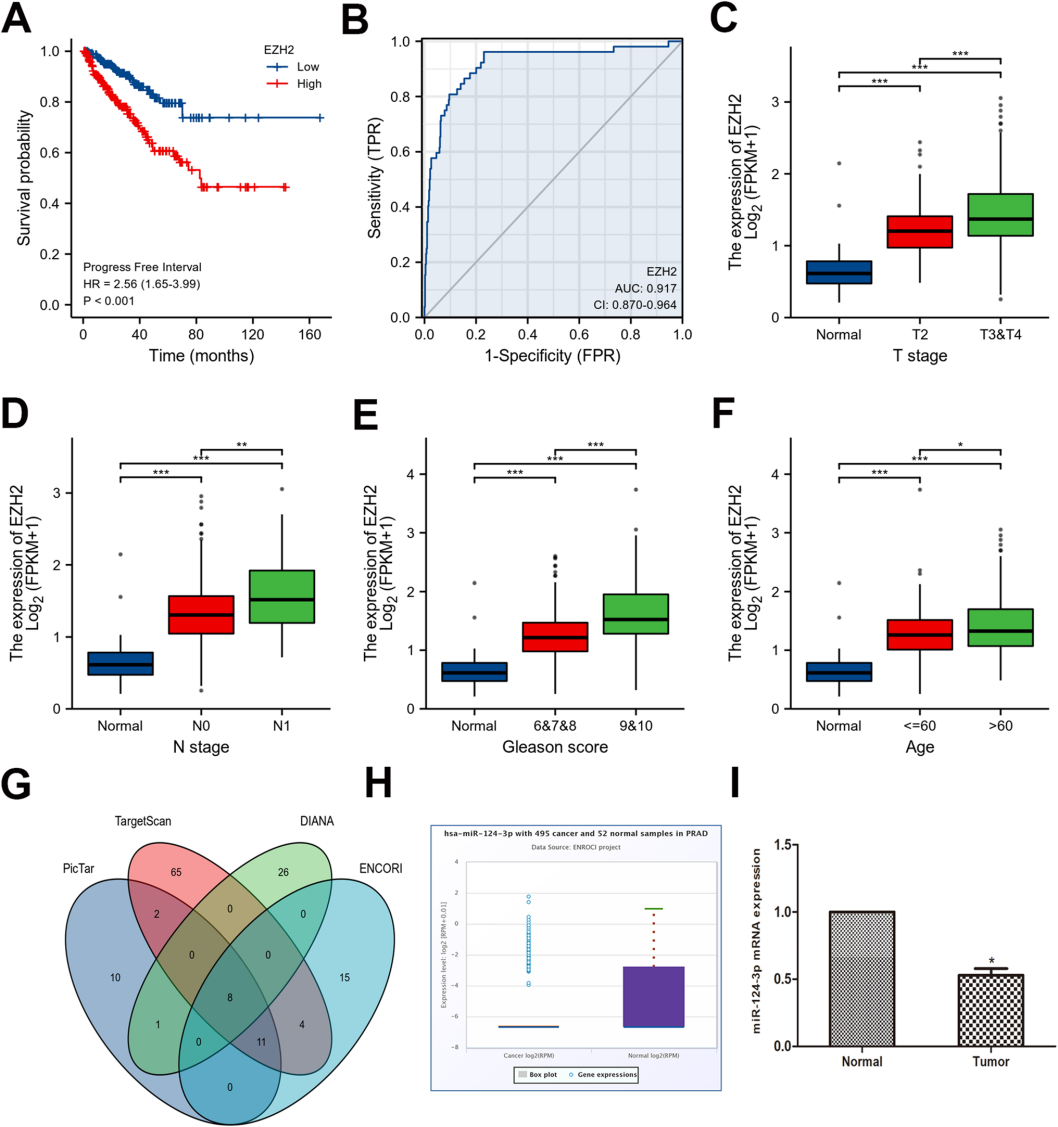
Prognostic and diagnostic value of EZH2 in PCa patients in TCGA along with potential regulatory miRNAs of EZH2. The data were retrieved from the TCGA database, encompassing 499 cancerous samples and 52 normal prostate samples. **A** PFI curve of different EZH2 expression levels in PCa. **B** Diagnostic ROC curve for distinguishing PCa tissues from normal tissues. EZH2 expression is considerably linked to **C** T stage, **D** N stage, **E** Gleason score, and **F** age. **G** EZH2 potential regulatory miRNAs intersection anticipated by the four databases were chosen using a Venn diagram. **H** ENCORI platform revealed a reduced miR-124-3p content in PCa samples in contrast with that in matching non-malignant samples. **I** Assessment of relative miR-124-3p levels in tumor tissues paired with neighboring non-malignant tissues (*p < 0.05; **p < 0.01; ***p < 0.001; ns, no significant difference)

The data with corresponding clinical features were retrieved from the TCGA database, encompassing 499 cancerous samples and 52 normal prostate samples. Based on the Kaplan-Meier survival curve, PCa patients with elevated EZH2 levels demonstrated a lower progression-free interval (PFI) (Fig. 2A). To assess the efficiency of EZH2 mRNA levels in differentiating PCa tissues from normal tissues, ROC analysis was performed, which estimated an AUC of 0.917 (Fig. 2B). Associations between high EZH2 mRNA levels and higher T stage, N stage, Gleason score, and age were significant (Fig. 2C–F). The possible regulatory miRNAs of EZH2 were predicted through bioinformatics analysis (PicTar (Krek et al., 2005), TargetScan (McGeary et al., 2019), DIANA (Paraskevopoulou et al., 2013), and ENCORI (Li et al., 2014)), and the intersections were determined. Eight overlapping miRNAs were screened: miR-124-3p, miR-144-3p, miR-101-3p, miR-25-3p, miR-26a-5p, miR-217, miR-138-5p, and miR-92b-3p. Common predictions from the four databases were utilized to obtain miR-124-3p for subsequent investigations (Fig. 2G). The ENCORI database illustrated that miR-124-3p was remarkably decreased in PCa tissues in contrast with nonmalignant tissues (Fig. 2H). Reduced miR-124-3p levels in tumor tissues were confirmed by measuring miR-124-3p levels in human tissues (Fig. 2I).

### MiR-124-3p directly targets EZH2 and inversely modulates EZH2 expression

**Fig. 3:**
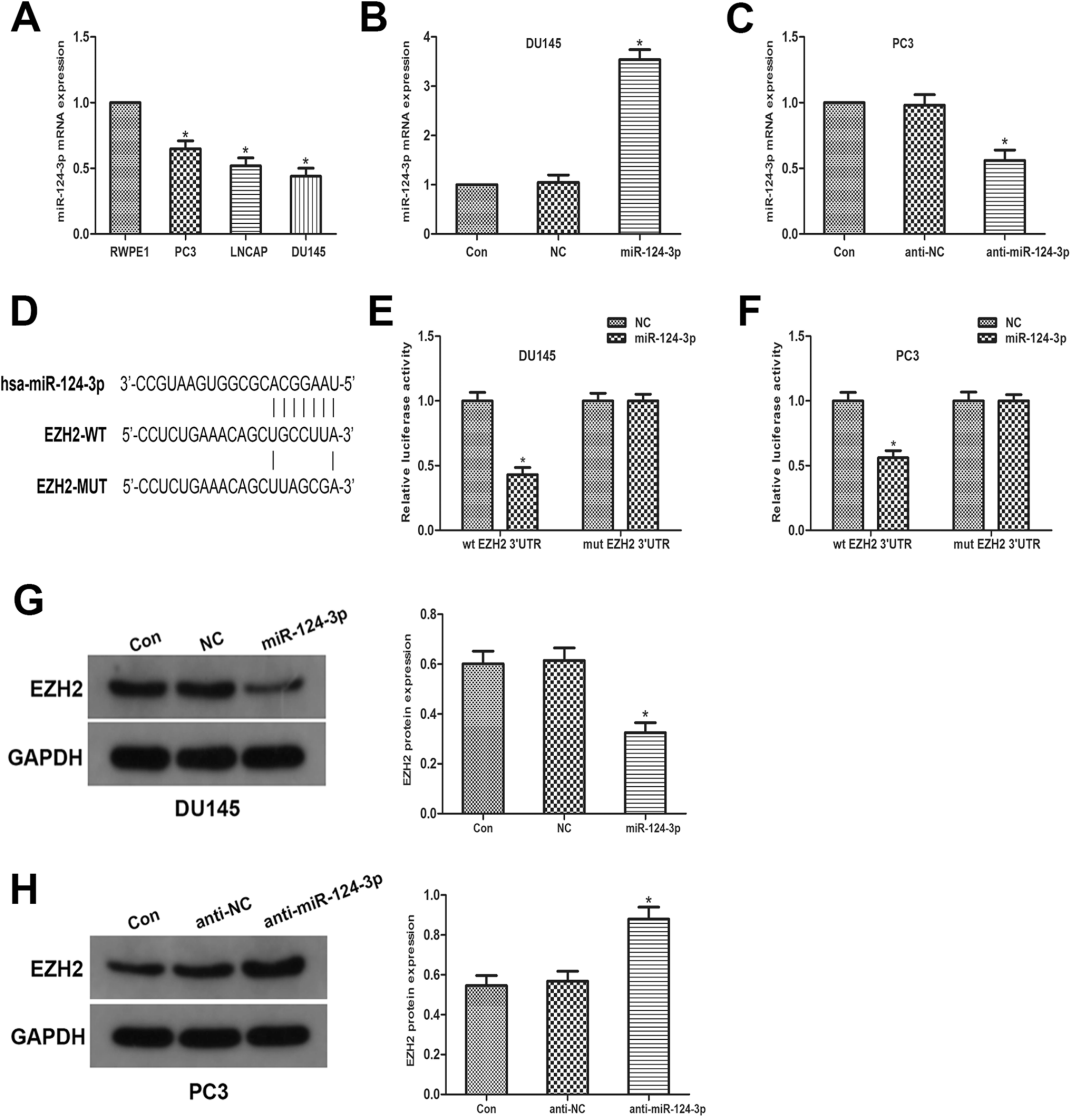
MiR-124-3p directly targets EZH2 and inversely modulates EZH2 expression. **A** MiR-124-3p content in RWPE1, LNCaP, PC3, and DU145 cell lines. **B**, **C** Transfection of DU145 cells and PC3 cells with miR-124-3p analogs or inhibitors. **D** The anticipated miR-124-3p docking sites sequence alignment in EZH2 3′UTR, as well as its altered sequence for the luciferase enzyme reporter test. **E**, **F** For DU145 and PC3 cell co-transfects of miR-124-3p analogs and reporter vectors carrying the mutant EZH2 3'UTR or EZH2 3′UTR, a luciferase enzyme reporter test was done. The luciferase enzyme activities comparison is given. **G**, **H** EZH2 expression Western blotting assessment in miR-124-3p repressors or analogs in PC3 cells transfects (*p < 0.05 vs. NC inhibitors)

EZH2 and miR-124-3p correlations were validated by first exploring the miR-124-3p content in PCa cell lines (DU145, LNCap, and PC3), as well as in the RWPE-1 human prostate epithelial cell line. The qRT-PCR analysis data demonstrated miR-124-3p content downregulation in PCa cells, contrary to the non-malignant cells (Fig. 3A). PC3 and DU145 cells were used in further experiments. The cells were transfected with miR-124-3p analogs or repressors to obtain miR-124-3p overexpressing or silenced cells (Fig. 3 B and C). Using open-access software, including miRDB, TargetScan, ENCORI, and DIANA, we hypothesized that miR-124-3p is an EZH2 upstream modulator and determined a docking site for miR-124-3p in the EZH2 3'UTR (Fig. 3D). This hypothesis was validated using a luciferase reporter gene test. The findings revealed that the EZH2 3′UTR reporter luciferase activity in miR-124-3p-overexpressing cells was considerably reduced; however, this effect was reduced by a mutation within the EZH2 3′UTR docking site (Fig. 3 E and F). Using western blotting, we examined the EZH2 content. The data revealed a significant decline in EZH2 content in miR-124-3p-overexpressing cells and a moderate elevation in miR-124-3p-silenced cells (Fig. 3G–H). These results indicate that miR-124-3p targets EZH2 directly to suppress its expression.

### MiR-124-3p modulates PCa cell growth, apoptosis, and invasion

**Fig. 4:**
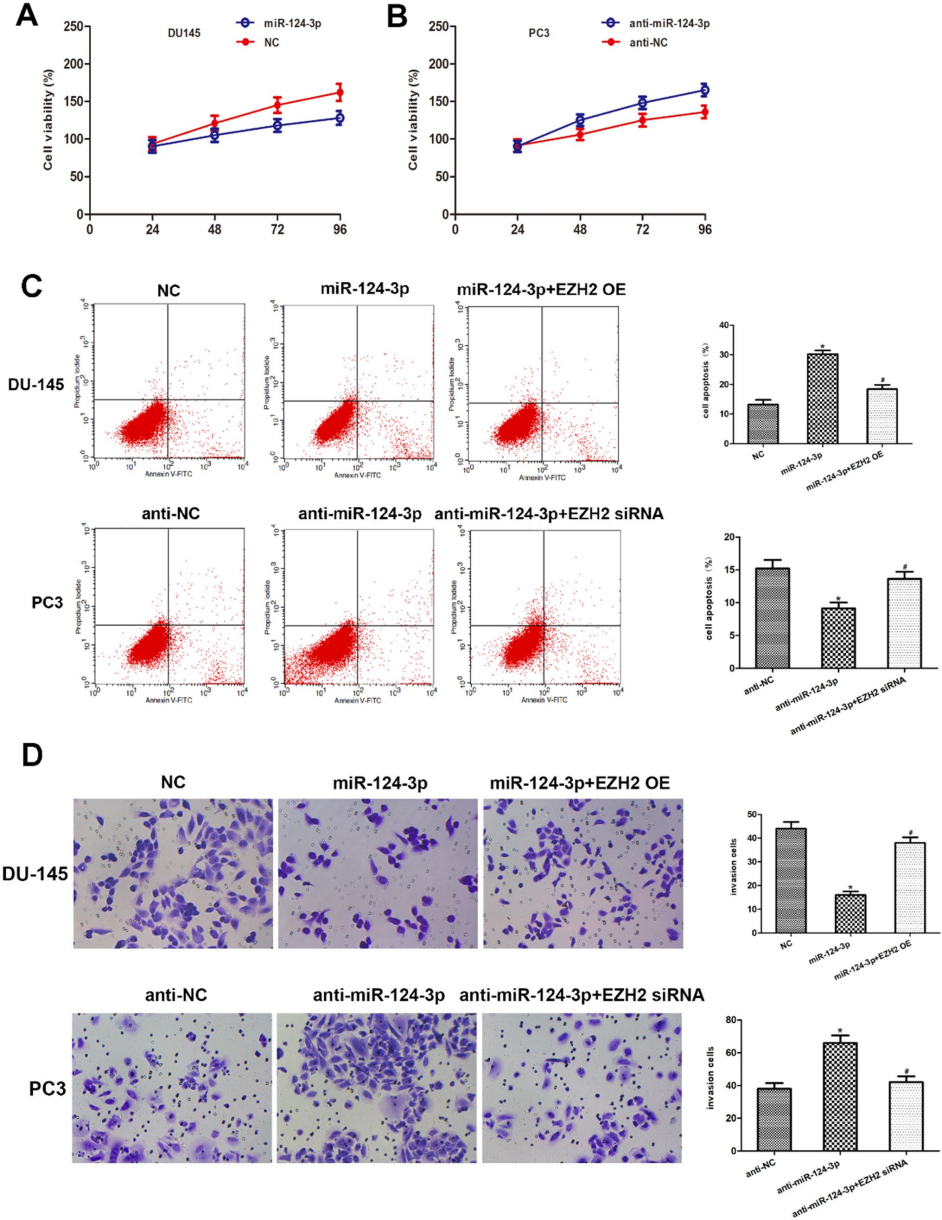
MiR-124-3p modulates PCa cell growth. **A**, **B** Cell viability in miR-124-3p repressors or analogs in PC3 cell transfects investigated using an MTT test. At 24, 48, 72, and 96 h after transfection, the absorbance values were measured. **C** Flow cytometry was done 48 h post-transfection. The histogram shows the apoptotic cell rate. **D** DU145 and PC3 cell miR-124-3p repressors or analogs transfect along with overexpression of EZH2 or knockdown of EZH2 tested in Transwell infiltration tests utilizing Matrigel-coated membranes to demonstrate infiltrative abilities (*p < 0.05 vs. NC inhibitors)

**Fig. 5:**
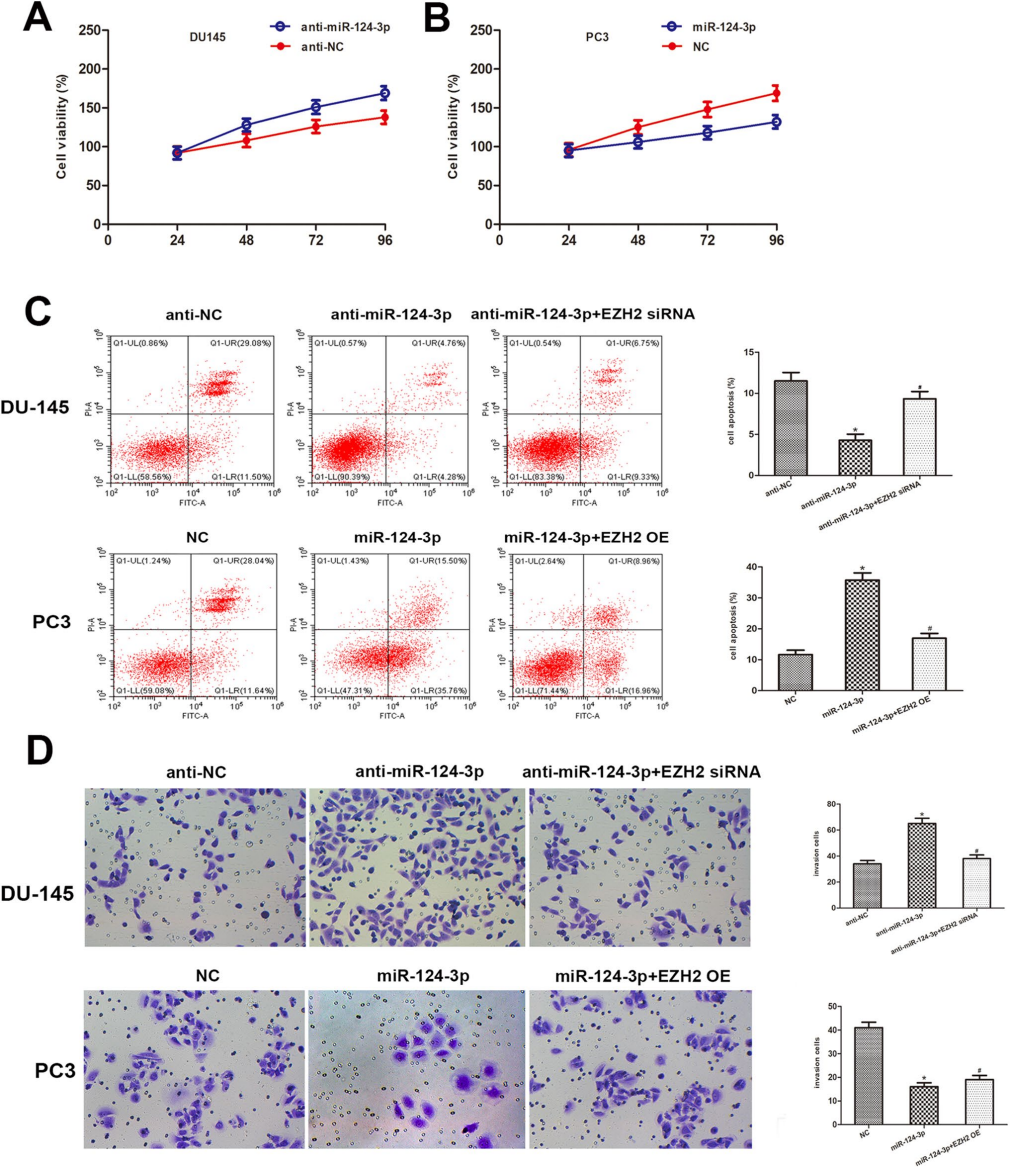
MiR-124-3p modulates PCa cell growth. **A**, **B** Cell viability in miR-124-3p repressors or analogs in DU145 cell and PC3 cell transfects investigated using an MTT test. At 24, 48, 72, and 96 h after transfection, the absorbance values were measured. (C) Flow cytometry was done 48 h post-transfection. The histogram shows the apoptotic cell rate. (D) DU145 cell and PC3 cell miR-124-3p repressors or analogs transfect along with overexpression of EZH2 or knockdown of EZH2 tested in Transwell infiltration tests utilizing Matrigel-coated membranes to demonstrate infiltrative abilities (*p < 0.05 vs. NC inhibitors)

The miR-124-3p function in PCa cell growth was investigated by conducting an MTT assay, revealing that DU145 cell and PC3 cell transfects of miR-124-3p analog viability were considerably reduced compared with the controls, and the miR-124-3p inhibitor cell transfects significantly promoted DU145 cell and PC3 cell viability (Fig. 4 A and B, Fig. 5 A and B). In addition, the function of miR-124-3p and EZH2 in PCa cell apoptosis was evaluated. Flow cytometry data revealed that miR-124-3p overexpression led to a significantly high percentage of PCa cell apoptotic nuclei, and miR-124-3p inhibition led to a significantly lower percentage of PCa cell apoptotic nuclei. The restoration of EZH2 expression significantly ameliorated the miR-124-3p induced promotion of PCa cell apoptosis (Fig. 4C, Fig. 5C). Taken together, these data revealed that miR-124-3p may affect the growth and apoptosis of PCa cells through the regulation of EZH2 expression. The effects of miR-124-3p on PCa cell infiltrative potential were then investigated. MiR-124-3p upregulation suppressed DU145 cell and PC3 cell invasion, whereas miR-124-3p downregulation boosted DU145 cell and PC3 cell invasion, as displayed by the Transwell assay. The restoration of EZH2 expression significantly ameliorated the miR-124-3p induced suppression of PCa cell invasion (Fig. 4D, Fig. 5D).

### The miR-124-3p effects on AKT pathway in PCa cells

**Fig. 6:**
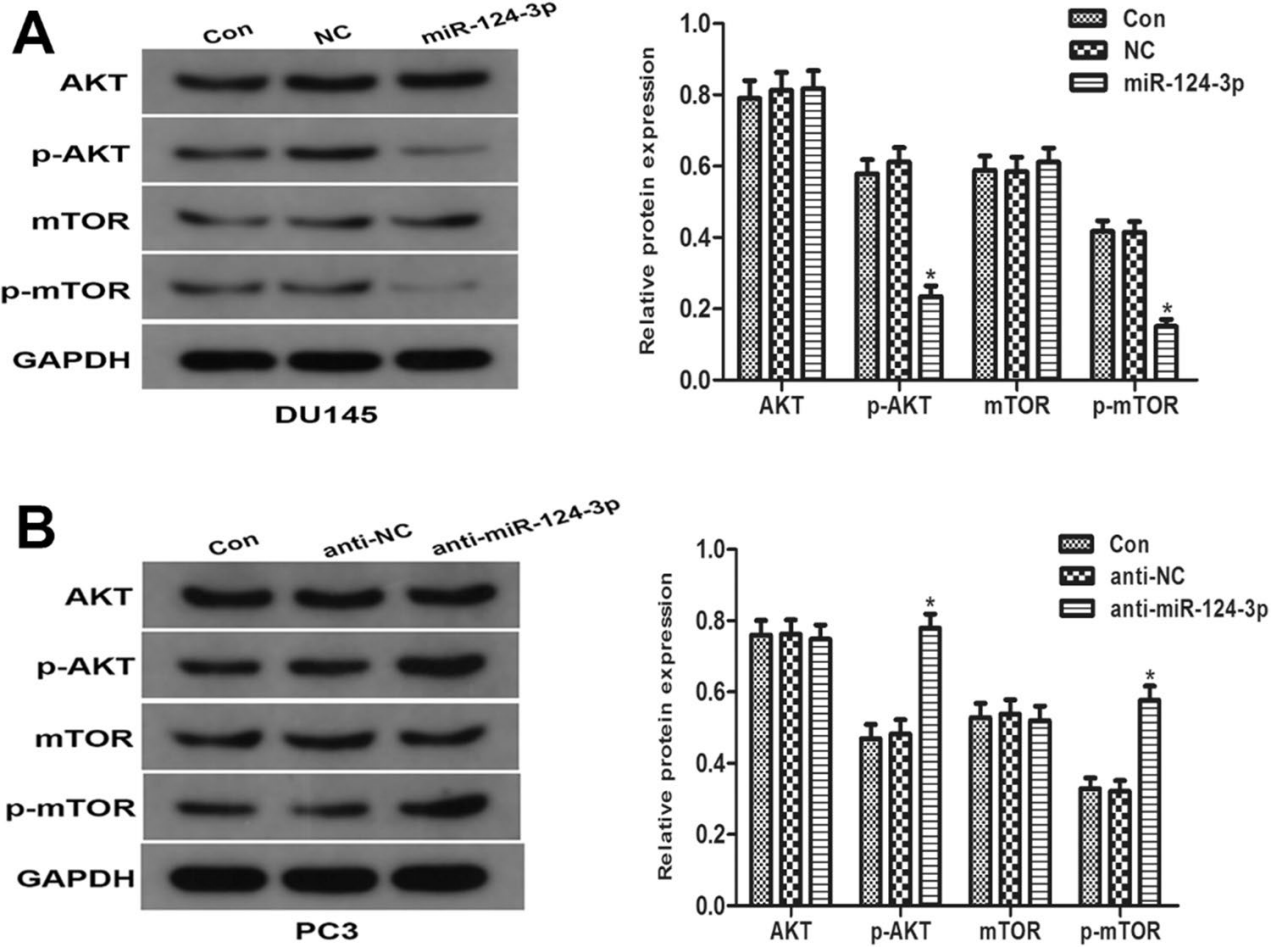
The miR-124-3p impacts on AKT pathway in PCa cells. AKT, p-AKT, mTOR, and p-mTOR protein contents in miR-124-3p repressors or analogs in PCa cell transfects assessed by Western blotting (Fig. 6**A**, **B**). The standardization control was GAPDH (*p < 0.05 vs. NC inhibitors)

The miR-124-3p repressors or analogs were transfected into PCa cells (DU145, PC3) to study the miR-124-3p impact on PCa proliferation. Western blotting data revealed that miR-124-3p overexpression in DU145 cells decreased p-AKT and p-mTOR content (Fig. 6A), while miR-124-3p inhibitors in PC3 cells increased these levels (Fig. 6B). These findings demonstrate that miR-124-3p could act as a key modulator by the AKT pathway.

## Discussion

Prostate cancer is highly prevalent, with an estimated three million men living with the disease in the USA. It is the second leading cause of cancer-related mortality in developed countries (Kargbo, 2019; Zhou et al., 2017). The usual therapy for advanced prostate cancer is androgen deprivation therapy (ADT), in addition to surgery and radiotherapy. Enzalutamide, bicalutamide, and abiraterone are some of the most commonly used drugs (Ramnarine et al., 2018). Despite major advancements in diagnosing and treating PCa, the long-term prognosis or five-year overall survival of patients with PCa remains poor (Wei et al., 2020). As a result, it is extremely important to investigate useful biomarkers and probable molecular pathways in patients with PCa to improve treatment (Chen et al., 2020a).

EZH2 is abnormally expressed in various cancers, boosting cancer cell motility, invasion, and proliferation via several routes (Zhang et al., 2021). Furthermore, current research has demonstrated a correlation between PCa and EZH2. EZH2 in PCa cells may hasten tumor development and act as a tumor oncogene (Jin et al., 2021). A miR-124-3p and EZH2 docking linkage was discovered using accessible bioinformatics data sources. Therefore, miR-124-3p was selected as the main miRNA for further investigation. In prostate cancer specimens, EZH2 expression was inversely linked to miR-124-3p in the current study. In contrast to paired non-malignant tissues, miR-124-3p levels were considerably lower in PCa tissues, whereas EZH2 was significantly elevated in tumors. A luciferase reporter assay was then used to determine whether EZH2 is directly affected by miR-124-3p. These findings indicate that miR-124-3p regulates EZH2 in PCa cells in a reversed manner, which is consistent with clinical specimen data.

miRNAs are implicated in several biological processes, such as cell growth, cell death, and differentiation. miR-124-3p expression in malignancies such as lung and human colorectal cancer has been documented (Chen et al., 2020b; Qiu et al., 2015). Dysregulation of miRNA-controlled physiological homeostasis has recently been linked to PCa carcinogenesis (Massillo et al., 2017). Nonetheless, the clinical importance of miR-124-3p and its biological involvement in PCa progression remain unknown. PC3 cells were used in our in vitro studies to induce PCa progression. The findings revealed that suppressing miR-124-3p in PCa cell lines increased cell proliferation and infiltration and decreased apoptosis, whereas miR-124-3p overexpression had the opposite effect. Our findings imply that miR-124-3p could be exploited as a predictive biomarker and therapeutic target for PCa.

The importance of the AKT/mTOR signaling pathway in disease development has become clear. In prostate tumor cells, the current research results stated that knocking down miR-124-3p led to a considerable reduction in AKT and mTOR levels, whereas overexpressing miR-124-3p had the opposite effect. We plan to further investigate the miR-124-3p effect on the AKT/mTOR pathway regulation in future studies.

## Conclusion

The research findings demonstrated that miR-124-3p could play a pivotal role in PCa cell carcinogenesis by cell proliferation, infiltration, and promotion of apoptosis inhibition via the AKT/mTOR pathway, which could be attributed, at least in part, to the inverse regulation of EZH2.

## Data Availability

The datasets used and analyzed during the current study are available from the corresponding author on reasonable request.
